# Impact of pulmonary rehabilitation on patients with different chronic respiratory diseases during hospitalization

**DOI:** 10.1097/MD.0000000000037778

**Published:** 2024-04-12

**Authors:** Xin-Yu Shi, Yi Ren, Xiao-Meng Gu, Yan-Rui Jia, Xue Wang

**Affiliations:** a Department of Respiratory and Critical Care Medicine, Beijing Institute of Respiratory Medicine and Beijing Chao-Yang Hospital, Capital Medical University, Beijing, China.

**Keywords:** inpatients, pulmonary rehabilitation, respiratory disease

## Abstract

The impact of pulmonary rehabilitation (PR) on patients with different chronic respiratory diseases (CRDs) during hospitalization has not been thoroughly evaluated before. The objectives of the current research were to assess the effect of comprehensive PR management on inpatients’ self-management skills, exercise capacity, nutrition assessment and mental health issues and explore whether impacts of PR vary in different CRDs. This retrospective study analyzed the clinical data from 272 inpatients with CRDs receiving PR management during hospitalization between October 2020 and March 2022 in Beijing Chao-Yang Hospital. Significant improvements were found in the patients’ ability of daily living (ADL), dyspnea (assessed by modified medical research council dyspnea scale (MMRC)), handgrip strength, maximal inspiratory and expiratory pressure, anxiety (using the 7-item generalized anxiety disorder scale (GAD-7)) and depression (the 9-item patient health questionnaire score (PHQ-9)). There was no significant change in nutrition assessment pre-post PR management during hospitalization. The subgroup analyses were conducted on hospitalized patients with chronic obstructive pulmonary disease (COPD), bronchiectasis, asthma, interstitial lung diseases (ILDs) and other CRDs (e.g., lung cancer, diaphragm hemiparesis, obesity, etc.). The results showed that ADL, MMRC score, MIP, MEP, PHQ-9 score improved in all subgroups with CRDs. Handgrip strength of left hand was increased in COPD inpatients and anxiety was improved in all subgroups except for ILDs. Comprehensive PR management was necessary and beneficial for patients with different CRDs during hospitalization.

## 1. Introduction

Chronic respiratory diseases (CRDs) stand as prominent contributors to global morbidity and mortality.^[1,2]^ Patients afflicted with CRDs, such as chronic obstructive pulmonary disease (COPD), interstitial lung disease (ILD), bronchiectasis, asthma, often experience recurrent hospitalizations, reduced life quality and expectancy, decreased exercise tolerance and a heightened likelihood of anxiety and depression.^[3]^ Clinically, hospitalized patients experiencing acute exacerbation are primarily treated to improve ventilation function, often overlooking the systemic manifestations of respiratory diseases, encompassing inadequate self-management skills, peripheral muscle dysfunction, maladaptive coping skills, and nutritional depletion which are common complex comorbidities typically present in these patients.^[4,5]^ It is known that pulmonary rehabilitation (PR) could ameliorate symptoms such as dyspnea and fatigue, enhance exercise tolerance and life quality, alleviate the burden on healthcare resources, and increase physical activity in patients with COPD.^[6,7]^ Nowadays, there is substantial and accumulating evidence indicating that PR has equivalent benefits in CRDs besides COPD, such as asthma, bronchiectasis, cystic fibrosis, lung cancer, pulmonary hypertension and ILDs.^[8–10]^

In the world, PR programs exhibit variations in their content and organizational aspects across different countries.^[11,12]^ Most of PR programs are offered in clinics targeting discharged patients or outpatients, which is likely formulated based on local conditions rather than evidence-based medicine research outcomes.^[13,14]^ The necessity of a progressive, comprehensive rehabilitation intervention for inpatients with CRDs during their hospitalization period warrants significant attention. While the impacts of PR have been evaluated by several previous studies, it has not been assessed in the context of hospitalized patients with various CRDs before.^[15–17]^ Consequently, the objectives of the present study were to evaluate the impacts of comprehensive PR management on inpatients’ exercise capacity, self-management skills, nutrition and mental status, and to investigate potential variations in these impacts among different CRDs.

## 2. Methods

### 2.1. Inclusion and exclusion criteria

We retrospectively reviewed the clinical data of 272 patients who accepted comprehensive PR management during hospitalization in Department of Respiratory and Critical Care Medicine, Beijing Chao-Yang Hospital from October 2020 to March 2022. Inclusion criteria of hospitalized patients are listed as follows: age ≥* *18 years old; ability to receive and understand exercise information; approval from respiratory physician; confirmed diagnosis of chronic respiratory disease. Exclusion criteria: inability to receive and understand exercise information; inability to take care of self; severe uncontrollable pain; significant cardiac diseases. This protocol had been approved by the Institutional Review Board of Beijing Chao-Yang Hospital.

### 2.2. Pulmonary rehabilitation program

Patients accepted comprehensive PR management developed by PR team for no less than 30 minutes twice a day. Comprehensive PR management included airway clearance techniques (e.g., airway oscillation, external oscillation, postural drainage, active cycle of breathing techniques, airway atomization, selection of airway temperature and humidification, etc.), respiratory function exercise (e.g., guide patients to conduct inspiratory muscle training, controlled breathing techniques, diaphragmatic breathing training; develop a training plan of respiratory resistance according to the actual measurement situation, etc.), and physical training (e.g., instruct patients to carry out upper limb resistance training, lower limb treadmill, and aerobic breathing training exercises, etc.). The intervention plan was designed around the needs of individual patients and their “treatable traits”^[18]^ and was selected, arranged and combined according to the individual conditions of patients by PR team so as to formulate reasonable comprehensive PR strategies.

### 2.3. Measurement of outcome variables

Outcome variables were measured and assessed at the beginning and end of the PR management during hospitalization. Ability of daily living (ADL) was assessed using ADL scale (overall scores range 0 to 100, where 0 = “absolute dependence”). Dyspnea was evaluated by means of modified medical research council dyspnea scale (scores range 0–4, 4 = “worst possible dyspnea”). Handgrip strength was measured using a grip dynamo-meter. Maximal inspiratory pressure (MIP) and maximal expiratory pressure (MEP) were gauged by a handheld mouth pressure meter. MIP and MEP were measured at least 5 times, no more than 8 times, until the variation rate was less than 10% for 3 consecutive maneuvers.^[19]^ Also, the assessment of anxiety and depression was conducted by the 7-item Generalized anxiety disorder scale^[20]^ and the 9-item Patient health questionnaire (PHQ-9),^[21]^ respectively. Nutrition assessment was carried out by measurement of arm and abdominal circumference, skin-fold thickness of upper arm and abdomen and mini-nutritional assessment (MNA) scale (MNA < 17 means definite malnutrition).

### 2.4. Data analysis

The baseline characteristics and outcome variables of the participants included in the PR program were presented by descriptive statistics. Normality was examined by the Kolmogorov–Smirnov test. Means (standard deviations (SDs)) were used for continuous variables while percentages were used for categorical variables. We performed paired t-tests to examine mean differences in self-efficacy, exercise tolerance, nutrition assessment as well as mental health status before and after the PR program. All statistical analyses were performed using IMB SPSS Statistic for Windows, version 24.0 (IBM Corp., Armonk, NY) and *P* values ≤ .05 was considered statistical significance.

## 3. Results

### 3.1. Characteristics of the participants

272 inpatients with CRDs accepted PR interventions between 2020 and 2022, of which 83 were women. The mean age of the population was 65.2 years old. 131 patients had COPD, 44 had asthma, 73 had bronchiectasis, 44 had ILDs, 37 had other CRDs (e.g., lung cancer, diaphragm hemiparesis, obesity, etc.). Twenty-eight percent of the patients had more than one chronic respiratory disease simultaneously (Table 1).

**Table 1:** Characteristics of the hospitalized patients who completed the PR program. (N = 272).

Characteristics	N (%)
Gender	
Female	83 (30.5)
Male	189 (69.5)
Age, Mean years (SD)	65.2 (13.1)
Lung diseases	
COPD	131 (48.2)
Asthma	44 (16.2)
Bronchiectasis	73 (26.8)
ILDs	44 (16.2)
Other lung diseases	37 (13.6)

COPD = chronic obstructive pulmonary disease, ILD = interstitial lung disease, PR = pulmonary rehabilitation.

* Participants may have more than one respiratory condition.

### 3.2. Improvement of outcome indicators

At the end of the PR program, the score of ADL increased while the dyspnea scale decreased in all the participants (Table 2). The grip strength of left hand and right hand increased with 1.4 and 1.3 in several; MIP and MEP increased with 6.4 and 9.1, respectively; 7-item Generalized anxiety disorder scale score decreased with 1.4; PHQ-9 score decreased by 1.8. There was no significant change in nutrition assessment rating score including arm circumference, abdominal circumference, upper arm and abdominal skin-fold thickness and the MNA scale score pre and post PR management.

**Table 2:** Change in outcome variables of participants pre and post the PR program.

Outcomes	All participants			Change	*P* value
	N	Pre Mean (SD)	Post Mean (SD)	Mean (SD)	
ADL	243	78.8 (22.1)	86.0 (17.8)	7.2 (14.0)	<.001*
MMRC dyspnea scale	270	2.6 (1.5)	2.0 (1.9)	−0.6 (2.0)	<.001*
Grip strength of left hand	268	22.2 (9.1)	23.9 (9.0)	1.4 (5.8)	<.001*
Grip strength of right hand	267	23.6 (10.4)	24.9 (10.1)	1.3 (6.3)	.007*
Maximal inspiratory pressure	272	46.5 (24.6)	52.9 (25.1)	6.4 (12.9)	<.001*
Maximal expiratory pressure	272	58.8 (28.2)	67.9 (32.8)	9.1 (18.2)	<.001*
Generalized anxiety disorder scale-7	272	3.4 (3.9)	2.0 (3.1)	−1.4 (3.5)	<.001*
Patient health questionnaire-9	272	5.4 (5.3)	4.0 (4.2)	−1.8 (4.5)	<.001*
Arm circumference	272	20.5 (8.1)	20.5 (7.7)	0 (3.8)	.443
Abdominal circumference	272	25.9 (3.8)	25.9 (5.0)	0 (6.4)	.145
Upper arm skinfold thickness	272	26.7 (11.6)	25.7 (10.1)	−1.1 (4.2)	.148
Abdominal skinfold thickness	272	88.4 (14.3)	87.7 (13.6)	−0.7 (7.0)	.094
Mini Nutritional Assessment scale	156	13.6 (6.5)	14.4 (5.9)	0.8 (6.2)	.543

ADL = ability of daily living, MMRC = modified medical research council, PR = pulmonary rehabilitation.

* Statistically significant between 2 groups.

### 3.3. Subgroup analysis of different chronic respiratory diseases

**Figure 1.**
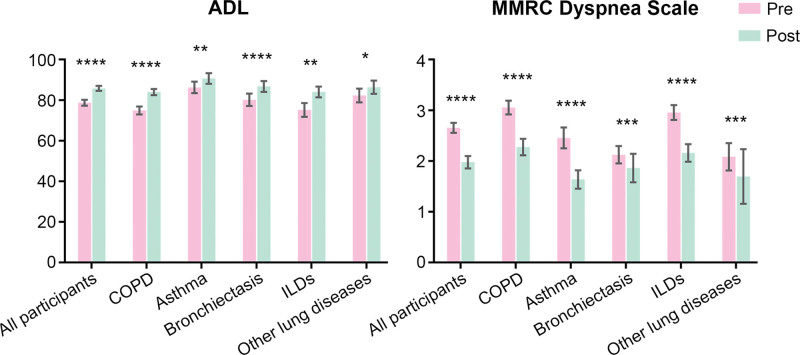
Scores of ADL and MMRC dyspnea scale pre-post PR program. ADL = ability of daily living, MMRC = modified medical research council.

**Figure 2.**
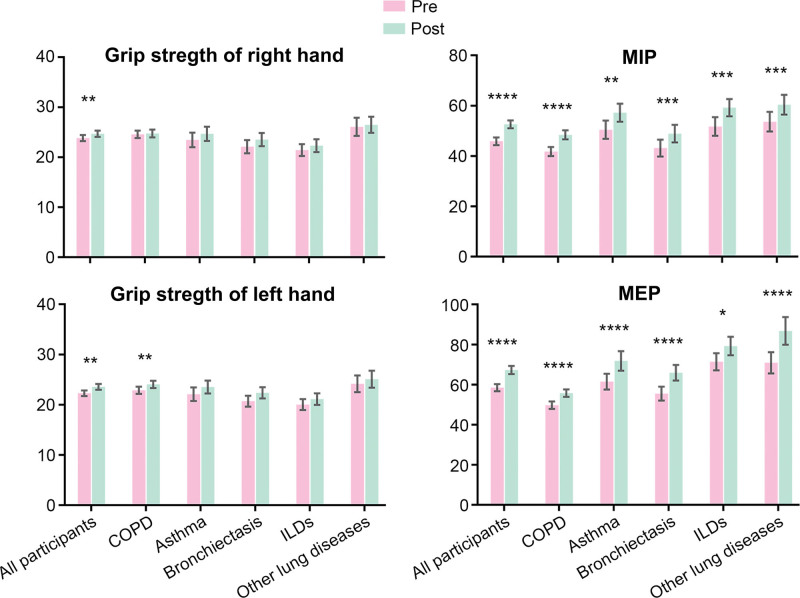
Grip strength and maximal respiratory pressure pre-post PR program. PR = pulmonary rehabilitation.

**Figure 3.**
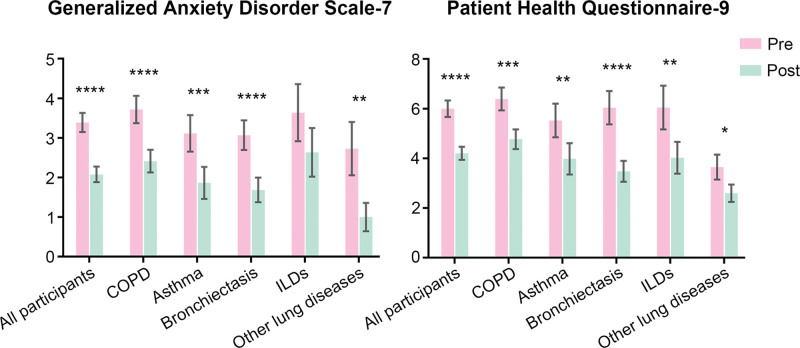
Assessment of depression and anxiety pre-post PR program. PR = pulmonary rehabilitation.

**Figure 4.**
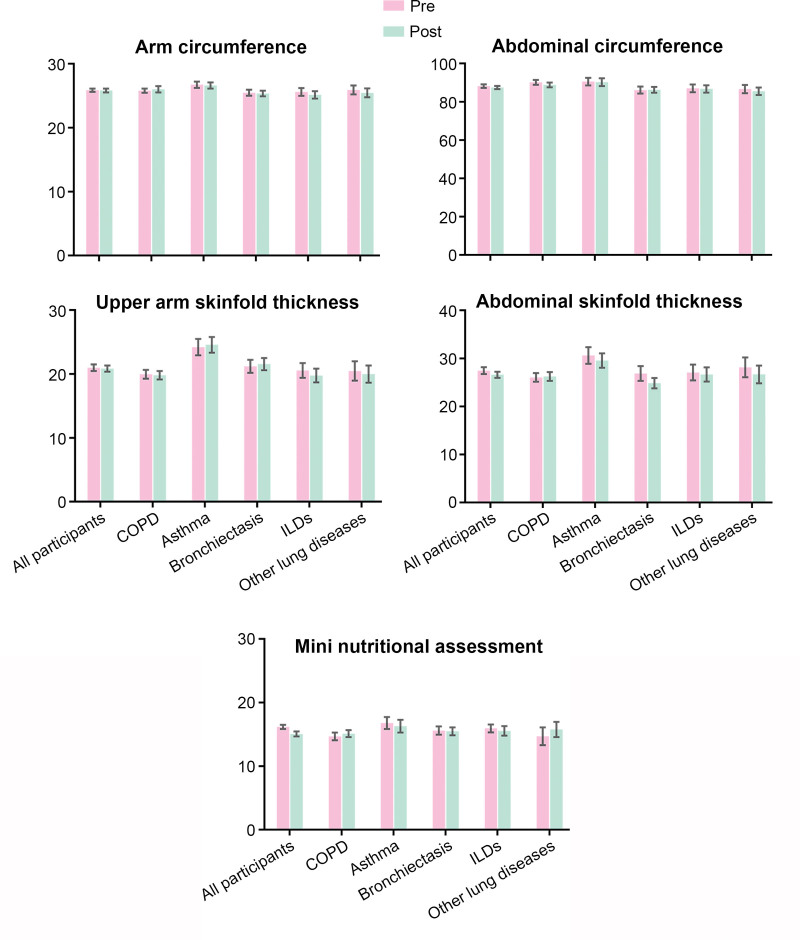
Nutrition assessment pre-post PR program. PR = pulmonary rehabilitation.

The results of subgroup analyses of different CRDs are presented in Figures 1–4. ADL score increased and dyspnea improved in all inpatients’ subgroups of the chronic respiratory disease (Fig. 1). All the patients showed significant improvements in MIP and MEP. Grip strength of left hand only increased in inpatients with COPD (Fig. 2). Depression was improved in all patients and except for patients with ILDs, the participants showed significant reduction in the anxiety assessment score (Fig. 3). There was no significant change in nutrition assessment including arm circumference, abdominal circumference, upper arm and abdominal skin-fold thickness, and MNA scale score pre and post PR program among all the subgroups (Fig. 4).

## 4. Discussion

Dyspnea, fatigue, decreased physical activity, peripheral muscle strength disorders are common characteristics of patients with CRDs and PR specifically targets these comorbidities. In addition, PR strives to ameliorate conditions that can lead to debilitation, including malnutrition, limited coping abilities, refuse to breathlessness-producing activities and mental health disorders.^[22,23]^

Despite the ideal scenario where PR is ought to be readily obtainable to all patients with CRDs besides optimal medical therapy, there exists a significant disparity between the demand for and the supply of PR interventions in numerous countries.^[14]^ A majority of PR studies have been conducted on outpatient populations, primarily focusing on COPD.^[16,24]^ In contrast, inpatients experiencing acute exacerbation often face challenges in engaging in exercise-based rehabilitation programs, which will lead to a refusal of PR management and an increased frequency of hospital readmissions. Targeted interventions within PR during hospitalization could pave the way to following PR in the clinic or community, ultimately resulting in reduced health care costs and fewer hospital admissions due to exacerbations.

Previous studies have demonstrated that even a brief hospital stay decreases exercise capacity and peripheral muscle strength, irrespective of age or initial functional status.^[25]^ Inspiratory muscle training significantly improves MIP and published studies have shown variable benefits in exercise capacity, dyspnea, and functional status. Although inspiratory muscle training, breathing training have been applied clinically as an important part of PR for many years, inspiratory muscle training has not been proven added benefit when combined with conventional PR.^[26–28]^ According to our results, MIP and MEP showed significant improvement after comprehensive PR management including respiratory muscle training.

Mental health issues aggravate the burden of CRDs.^[29]^ In the current study, depression was improved in all patients. Except for patients with ILDs, all participants showed significant reduction in the anxiety assessment score. The impact of PR on the improvement of mood disorders in different CRDs especially ILDs still needs further study. It is estimated that 20-35% COPD patients in stable stage have poor nutritional status.^[30]^ Although during hospitalization, there was no significant change in nutrition assessment pre and post PR program which was suspected to be related with short duration of intervention, special attention was supposed be paid to optimal nutritional intervention on underweight patients.

This paper still has many deficiencies and limitations. Firstly, it was not a large-sized multi-center study and this research is retrospective and it seems inevitable to have selection, exclusion, and recall related bias. Secondly, this study didn’t set up a control group to with alternative interventions as compare. Thirdly, due to the severe symptoms in the acute aggravation of CRDs, some important indicators, such as the 6-minute walking experiment, FEV1, FVC could not be measured for most patients. Finally, 28% of the patients had more than one CRD, which may have influenced the results for individual subgroup. Nonetheless, it is usual for patients to have multiple comorbidities in the actual clinical situation. It is the degree of disease complexity that dictates the type of intervention and setting for the highest likelihood of success.^[24]^ Therefore, we believed that our research is valuable on assessing the impact of PR on the inpatients with CRDs.

## 5. Conclusion

Comprehensive PR management has significantly improved self-efficacy, exercise tolerance and capacity, mental health issues in patients with CRDs during hospitalization. The results of the current research may help raise awareness of the importance of PR as a critical component in the treatment of inpatients with CRDs.

## Author contributions

**Data curation:** Xiao-Meng Gu.

**Funding acquisition:** Xin-Yu Shi.

**Methodology:** Xin-Yu Shi, Yan-Rui Jia.

**Project administration:** Yi Ren.

**Supervision:** Xue Wang.

**Validation:** Xin-Yu Shi.
